# Clinical and laboratory characteristics and follow up of 62 cases of ketotic hypoglycemia: a retrospective study

**DOI:** 10.1186/s13633-019-0066-9

**Published:** 2019-11-02

**Authors:** Paul Kaplowitz, Hilal Sekizkardes

**Affiliations:** 1 Division of Endocrinology and Diabetes, Children’s National Health System, 111 Michigan Ave NW, Washington, DC 20010 USA; 2 Eunice Kennedy Shriver National Institute of Child Health and Human Development, National Institutes of Health, Bethesda, MD USA; 3 University of Tennessee Health Science Center, LeBonheur Children’s Hospital, Memphis, TN USA

**Keywords:** Ketotic hypoglycemia, Hypoglycemia disorders, Children, Glycogen storage disease

## Abstract

**Introduction:**

Idiopathic ketotic hypoglycemia (KH) is the most common cause of hypoglycemia in non-diabetic children ages 0.5–6 years old and typically occurs after a period of poor food intake. There are no large studies looking at the value of common laboratory testing in children presenting with KH or how often other diagnoses are made.

**Objectives:**

To examine the clinical presentations and the value of laboratory testing done in a cohort of children clinically diagnosed with KH.

**Methods:**

Billing records were searched from 2008 to 2017 for patients seen by the endocrine service for “hypoglycemia, not otherwise specified”. Charts were reviewed to determine age, sex, presenting symptoms and testing ordered at the time of the consult. Through chart reviews after the event and parent phone calls, diagnoses other than idiopathic KH were searched.

**Results:**

Of 150 charts reviewed, 62 had sufficient information to make a clinical diagnosis of KH (32 males 30 females, mean age 2.9 years). Most had a history of gastrointestinal illness or prolonged fasting but 29% had no apparent precipitating event. Laboratory testing was quite variable and while low serum CO_2_ was seen in over half, no routine hormone testing, metabolic testing or supervised fasting resulted in a relevant diagnosis. We identified 4 out of 62 (6.5%) with relevant diagnoses which explained KH, including one child with failure to thrive found to have growth hormone (GH) deficiency and 3 by genetic testing, including one case of GSD type 9α, but all had atypical presentations.

**Conclusions:**

In the typical setting of a healthy 0.5–6 year-old child with an uncomplicated episode of KH following poor food intake and a normal exam including growth, hormonal and metabolic testing can safely be deferred. However, frequent recurrences and atypical features should prompt further investigation.

**Trial registration:**

Not needed for a retrospective chart review study.

## Introduction

Ketotic hypoglycemia (KH) is the most common cause of hypoglycemia presenting to the Emergency Department (ED) in healthy children between 6 months and 6 years of age [1, 2]. It is typically triggered by decreased oral intake due to gastrointestinal illness with vomiting and/or prolonged fasting. Early studies suggested that these children have a decreased ability to tolerate a prolonged fast, with low levels of gluconeogenic amino acids and release of ketone bodies from fat stores [3–5]. It is recognized the KH is usually idiopathic and thus a diagnosis of exclusion. While in most cases, idiopathic KH resolves after a few episodes by age 6, several serious hormonal and metabolic conditions, including cortisol and growth hormone (GH) deficiency and various forms of glycogen storage disease (GSD) can present with KH. A study which enrolled 164 selected children who had 2 or more episodes of KH and negative hormonal and metabolic testing, found by DNA testing that 20 (12%) had a GSD, mostly X-linked GSD type 9 α [6]. However, the frequency with which GSD and other diagnoses are later found in a more representative sample of children presenting with KH is unclear. Previous reports of idiopathic KH have only included 8–24 patients and as a group showed a male predominance [6]. In addition, there is uncertainty as to the optimal evaluation for the child presenting with one or a few episodes of KH triggered by poor intake. One recent review states that “Biochemical findings include low insulin levels, appropriate cortisol and GH levels, elevated free fatty acid and ketone levels, negative response to glucagon stimulation testing during fasting hypoglycemia, normal thyroid hormone levels, and reassuring metabolic panels which rule out other defects (including plasma acylcarnitine profile, urine organic acids, and serum amino acids). It is also important to exclude other conditions that may contribute to hypoglycemia such as metabolic defects, adrenal insufficiency, or GH deficiency.” [7]. Another report which reviewed 18 patients with KH seen in the ED (Emergency Department) concluded that “in its typical presentation (previously healthy 1-5 year old with normal growth and development, a first episode of symptomatic fasting hypoglycemia with ketonuria, without hepatomegaly and with resolution of symptoms upon administration of glucose), an extensive and overzealous work-up for endocrinopathy or inborn error of metabolism is not necessary.” [1].

Our objective was to examine the clinical presentation and different biochemical tests performed in a large sample of children presenting with KH over a 10-year period at a large urban children’s hospital. Through review of the medical record following the index case and follow-up phone calls to parents, we hoped to determine the frequency of any endocrine, genetic or metabolic diagnosis found later in patients who were initially diagnosed with idiopathic KH.

## Methods

This study was approved by our institutional review board. Through billing records, we obtained a list of all endocrine consults for hypoglycemia in children between ages 6 months and 6 years done between 2008 and 2017. The CPT code searched for, “hypoglycemia not otherwise specified (NOS)”, was 251.2 in ICD-9 and E16.2 in ICD-10 starting in October 2015. Any patient with a concomitant diagnosis of type 1 diabetes was excluded. Out of 150 consults, 62 patients (32 males, 30 females) had adequate information in the electronic medical record (EHR) to make a likely diagnosis of KH based on the endocrine provider’s chart note. In each case, the lowest blood glucose (BG) recorded was 55 mg/dL (3 mmol/L) or less. We searched for evidence of urine ketones or elevated serum β-hydroxybutyrate at the time of the event or during a fast. For many of the non-included consults, KH was considered possible but there was either no or inadequate documentation of low BG and urine ketones at the time of the event. Others had known hyperinsulinism, post-prandial hypoglycemia, or multiple chronic non-endocrine conditions. Patients not included had their EHR searched for later visits with endocrinology or genetic service to ensure that important hypoglycemia-related diagnoses were not missed. We did include 9 patients with no documented urine ketones or negative ketones performed greater than 12 h after the event if the history was typical for KH or an elevated serum β-hydroxybutyrate was found at the time of the episode or during a monitored fast. Four patients who met the inclusion criteria were excluded due to concomitant propranolol use, a known risk factor for hypoglycemia [8].

Of the 62 patients, 60% were seen in the inpatient setting and 40% as outpatients. For the outpatient visits, thorough documentation of a recent hospital admission (often at outside institutions) for hypoglycemia was required. Items included in the review of the index event included age, sex, symptoms at the time of presentation (mainly altered mental status or seizures), precipitating events, and any previous history of similar events. Height, weight and BMI percentiles were recorded. Laboratory results included lowest blood glucose (often done by an emergency medical technician or at an outside ED before arrival in the ED at our institution), presence of urine ketones, serum CO_2_, cortisol, GH, and insulin, and whether a fasting study was done. When fasting studies were done, they continued for 12 h or ended either when the blood glucose was < 50 mg/dL or serum β-hydroxybutyrate became elevated. Metabolic studies recorded included mainly serum amino acids and urine organic acids; carnitine profiles were performed inconsistently. The chart was then reviewed for further admissions or ED visits for hypoglycemia, what genetic testing was performed for inborn errors of metabolism and any other endocrine or metabolic diagnoses made subsequent to the index visit. To capture information not available in the medical record, we attempted to contact parents first by mail and then by phone, to learn if there were additional episodes of hypoglycemia seen at a hospital or treated at home, the total number of episodes, the child’s age at the last episode, and whether any additional diagnoses were made. Since the dates of the consults were between 2008 and 2017 and chart reviews were done in late 2018, the range of the follow-up duration was 1 to 10 years with a mean follow up of 5 years. Only descriptive statistics are reported.

## Results

At the time of the initial consult, the mean age of the patients was 2.9 ± 1.3 years, and 79% were between 1.5 and 5 years. There were 32 males and 30 females. There were 69% who were hospitalized at our institution, 18% who were hospitalized elsewhere, and 8 of 62 (13%) who had an ED visit only. The most common presenting symptom was lethargy or altered mental status (72%), with 20% presenting with a seizure and 8% presenting with a low blood glucose during an ED visit due to GI illness without hypoglycemic symptoms. About 1/3 had a clear GI illness usually with vomiting for at least a day, whereas 1/3 had a history of decreased food intake the day before or the day of the event with no GI illness. However, 29% had no history of a precipitating event which could explain hypoglycemia. A history of a previous similar episode was elicited in 38%.

The mean height percentile (after excluding 6 patients with growth-limiting diagnoses including GH deficiency, chondrodysplasia punctate, Smith-Magenis syndrome and congenital muscular dystrophy) was 48.9 ± 29%, the mean weight percentile was 41.4 ± 27%, and the mean BMI percentile was 46.5 ± 30, with 5 having a BMI < 5th percentile.

Laboratory testing performed was quite variable (Table 1). The mean lowest blood glucose recorded was 37.3 ± 10.4 mg/dl (2.1 ± 0.6 mmol/L) . 78% had electrolytes documented on admission and the mean CO_2_ was somewhat low at 17.5 ± 3.9 mmol/L, with 57% (27/47) being < 18 mmol/L, indicating that mild metabolic acidosis was common. Hormonal testing was inconsistently ordered, and sometimes done > 12 h after admission. Cortisol was done in 62%, with a mean value of 17.4 ± 9.8 μg/dL (480 ± 270 nmol/L); 84% (31/37) had a level of > 10 μg/dL (> 276 nmol/L). Insulin levels were done in 45% and in 23/27 were <  2 mIU/L, with none higher than 4.5 mIU/L. However, in the 4 with detectable levels, it seemed likely that they were ordered after IV glucose and/or feeding. Random GH levels were done in only 28, and 76% had a level of < 7 ng/dL. A monitored fasting test, done either during the index visit or in a few cases during a follow-up admission or after referral to another center, was done in 27%, and in no case were the results consistent with any diagnosis other than KH. Metabolic studies done were mainly plasma amino acids and urine organic acids (58%), with carnitine profiles done in a smaller number. The few abnormalities found were felt to be consistent with KH; in one case glutaric aciduria was suspected but ruled out after subsequent urine and microarray testing. The complete dataset used for this section can be found in Additional file 1.

**Table 1:** Laboratory studies done in patients with KH (*N* = 62)

Assessment	Obtained in % of patients	Results
Glucose	100%	Mean 37.3 ± 10.4 mg/dL (2.1 ± 0.6 mmol/L
Serum CO_2_	78%	Mean 17.8 ± 3.9 mmol/L < 18 mmol/L in 58%
Serum cortisol	61%	Mean 17.4 ± 9.8 μg/dL (480 ± 270 nmol/L) > 10 μg/dL (> 276 nmol/L) in 84%
Serum growth hormone	28%	< 7 ng/ml in 76%
Serum insulin	45%	< 2 mIU/L in 23; 2–4.4 mIU/L in 4
Fasting study	27%	None provided diagnosis other than KH
Plasma amino acids and urine organic acids +/− carnitine profile	58%	None were diagnostic

## Results of follow-up with parent phone calls and chart reviews

Through parent phone calls (*n* = 37) and more recent chart notes on patients still being followed by Endocrinology or Genetic service, we were able to obtain updated information on 41/62 patients. Chart reviews revealed that 19/62 patients had additional visits to our hospital ER or inpatient service for hypoglycemia. Parents contacted were asked if they were given a good explanation as to the cause of their hypoglycemia; only 58% answered yes, but 83% recalled they were given helpful advice on how to prevent and treat any further episodes. Over half (55%) of parents reported additional episodes and 12/22 of those parents reported episodes which were treated at home without the need for an ER visit or hospital admission. Four parents reported seeing endocrine or genetic providers at other institutions, but no new diagnoses were made. When asked to recall the number of hypoglycemic episodes after the endocrine consult and including documented episodes before, 11 patients had only one hypoglycemic episode; 10 had 2–3 episodes, 12 had 4–5 episodes and 4 had > 5 (in all 4 cases at least 10) episodes (mean 3.3 episodes). The mean reported age at the last episode was 3.9 years and was 0.8–1.8 years in 8; 2–4.8 years in 15; and 5–7 years in 16. Four patients continued to have episodes after age 6.

No patients initially diagnosed with KH were later diagnosed with primary or secondary adrenal insufficiency or hyperinsulinism. One child diagnosed with GH deficiency during an admission for hypoglycemia is described below. Another patient who was 1.3 years old and growing at the 27th %ile with some recent fall-off was suspected of having GH deficiency when she had a GH of 2.1 ng/ml after a 12 h fast (lowest glucose 51) done 2 days after the index episode. Six months later she underwent GH stimulation testing with a peak GH of 5.1 ng/ml. She was treated for 8 months with GH, but had several more episodes of hypoglycemia, so GH was stopped, and hypoglycemia resolved by age 3.5. It was concluded that she did not have true GH deficiency. Mention of genetic testing for GSD was found in 8 patients, which included one positive (see below) and 4 patients underwent whole exome screening with negative results.

## Cases with significant diagnoses made after the index episode

1. A male child was seen at age 6 after the family moved from another city. His first episode of KH was at age 1 after emesis all night. He had multiple further episodes of KH triggered by poor oral intake treated in the ED with IV glucose; metabolic evaluations were negative but genetic testing was deferred. Height and weight were close to the 50th %ile and there was no hepatomegaly. After the endocrine consult, he was referred to the Genetic service which started cornstarch, resulting in decreased episodes, but hypoglycemic episodes increased again around age 8. A GSD panel done at Baylor found a hemizygous variant in the PHKA2 (phosphorylase B kinase) gene c.1490G > A (p.R497Q) which was not found in the database but was in a region highly conserved during evolution; this was felt to be consistent with GSD type 9α.
2. A female child had her first episode of KH at age 2.4 precipitated by emesis for 2 days. Height was 41%ile but weight was 3rd%ile and BMI was < 2 SD. Her initial laboratory studies were atypical with Na 126 mmol/L, CO_2_ 8 mmol/L, pH 7.24, glucose 37 mg/dL (2.1 mmol/L), and BUN 39 mg/dL (139 mmol/L), indicating significant dehydration and metabolic acidosis. It took 2 days of IV fluids to clear the urine ketones. One year later she had a second episode triggered by emesis with similar test results. A GSD and liver panel sent to Baylor was negative. After a third episode 4 months later (age 3.8), she was referred to a center specializing in hypoglycemia for a fasting study which was consistent with KH. A hypoglycemia panel eventually revealed a pathogenic mutation in the gene SLC16A1 (also called MCT-1), encoding monocarboxylate transporter 1, which is critical for ketone utilization and has been found to be a rare cause of KH [9]. She now receives 2 tablespoons of cornstarch at bedtime with frequent checks of BG and ketones if she is not eating well and is doing better.
3. A 4-year old girl was seen as an outpatient for evaluation of 2 episodes of KH at ages 3 and 3.5. She had height − 3.1 SD, weight − 4 SD and BMI − 2.4 SD. Exam showed a prominent forehead. Her IGF-1 was normal. She continued to have mild episodes of hypoglycemia which did not require hospitalization. At a Genetic visit at age 5.6, metabolic tests done when the glucose was normal were negative, but Russell-Silver syndrome (RSS) was suspected on the basis of her growth and physical exam; testing confirmed loss of methylation at DMR1 with biparental inheritance of chromosome 7.
4. A 2 ½ year old boy was admitted for hypoglycemia after a prolonged fast. He had been evaluated in the past by GI for failure to thrive; height was − 3.2 SD with weight − 3.5 SD. Insulin at the time of admission was suppressed; GH and cortisol were ordered but not done due to inadequate sample. Three days later, his am cortisol was 9.3 μg/dL (257 nmol/L); free T4 and TSH were normal. During the admission, several BGs were in the 40–60 range. After a 12 h fast, his β-hydroxybutyrate was very elevated at 34.7 mg/dl (3.3 mmol/L) when his glucose was 40 mg/dL (2,2 mmol/L). When his insulin-like growth factor 1 (IGF-1) came back < 16 ng/ml and IGF-binding protein 3 was 0.5 μg/ml (normal for age 1.3–3.5), GH deficiency was suspected, and an MRI showed pituitary hypoplasia, no stalk, and an ectopic posterior pituitary. He was started on GH with an excellent response and hypoglycemia has not recurred.
5. A child with a single episode of KH at age 1.8 and no further episodes was diagnosed with type 1 diabetes at age 5.1. While it seems likely these 2 events are unrelated, there have been older reports of hypoglycemia in infancy preceding either abnormal glucose tolerance years later [10] or diabetes mellitus requiring treatment [11]. However, in most cases the onset of hypoglycemia was earlier than in our patient and tended to be recurrent.

## Discussion

This study is the largest retrospective case series of patients with KH as previous case series only had 8–24 subjects [6]. It confirms that children with KH typically present to EDs between 6 months and 6 years of age. The mean age of in our study of 2.9 years is similar another study [2] reporting a mean age of 2.6 years at presentation of 24 cases. Nearly all of our subjects, except those with other growth-limiting diagnoses, had normal height, weight, and BMI, in contrast to an earlier study which found many of their patients below the 10th %ile in height and weight [3]. In most patients a clear precipitating event was identified, usually a GI illness with emesis or poor intake or in fewer cases a longer than usual fast, but in 29% of cases there appeared to be no precipitating event. Thus, while decreased intake clearly increases the risk for KH in young children, the diagnosis should not be ruled out in children who by report had normal food intake the day before or the day of the event. We did not find the male predominance of cases reported by Brown et al. in their literature review [6], suggesting our sample did not contain a significant number of cases of GSD 9α, which is x-linked. We also confirmed that repeat episodes (in most cases 4 or fewer) are common but that it is unusual for them to continue beyond age 6, though we did find 4 patients who had continued episodes between the ages of 6 and 8. Parent interviews revealed that many of the episodes after the consult with endocrinology were managed by the parents at home, suggesting that counseling parents to avoid prolonged fasting and how to treat future episodes at home (which 83% of parents contacted said was helpful) was of value.

The larger question we hoped to address is the value of the extensive laboratory testing which is often done in children with KH for the purpose of finding hypoglycemia-related diagnoses. After a careful review of the EHR for further hospital visits for hypoglycemia and endocrine and genetic outpatient notes, we attempted to contact the parents of our subjects. Combining the EHR reviews and parent interviews we had reliable follow-up information on 41 of 62 patients. In 5 cases described in detail, new endocrine or genetic diagnoses were eventually made. One of those, onset of type 1 diabetes 3 years following a single KH episode, was likely unrelated. The child with GSD type 9α clearly merited genetic testing based on the frequency and persistence of repeated episodes between the ages of 1 and 8 even though there was no hepatomegaly. The child eventually diagnosed with a ketone utilization defect caused by a mutation in MCT-1 was atypical at her presentation as she was the only patient in our study who presented with severe metabolic acidosis (a feature of MCT-1) and dehydration. Only 1 other patient in our study had a serum CO_2_ of < 12 mmol/L and none were < 10. The child eventually diagnosed with Russell-Silver syndrome, which has a known association with hypoglycemia [12], could have been suspected based on her growth and the physical findings, especially the prominent forehead. Thus, the incidence of significant new findings in our cohort was 4/62 or 6.5%.

There was a great deal of variability in the amount of testing done during the index episode of KH. While electrolytes, serum cortisol and plasma amino acids and urine organic acids were performed in over half of the patients, fewer than half had growth hormone and insulin levels, only a few had β-hydroxybutyrate levels done (mostly during monitored fasting) and only 27% had fasting studies done. This likely reflects the different approaches of the providers doing the consults, with the more experienced endocrinologists ordering fewer tests when the clinical scenario appeared typical for KH. The classic critical sample studies, often but not always done at the time of arrival in the ED, did not result in any new diagnoses. Insulin levels are unlikely to be helpful in the setting of a child with poor intake presenting at 6 months to 6 years of age with non-recurrent hypoglycemia and ketonuria, and not surprisingly, most children had insulin levels below the assay lower range. Congenital hyperinsulinism rarely presents at ages when KH is common, and insulinomas very rarely occur in young children. In both cases, the hypoglycemia is nonketotic or hypoketotic due to the effect of insulin in suppressing fat breakdown and ketone formation. It should be emphasized that true hypoketotic hypoglycemia is almost always pathological.

GH deficiency can occasionally present with KH after poor intake as GH is one of the hormones which helps to maintain glucose homeostasis during a prolonged fast. However, by the age of 1.5–5 years, at which most children have their episodes, normal linear growth is generally sufficient evidence to exclude this diagnosis. Nonetheless, GH levels were often done at the time of the episode or in the 12 h thereafter, and were less than 7 ng/ml in 76%. This was not surprising in that studies have shown a high frequency of “subnormal” (i.e. < 7.5 ng/mL) GH responses to spontaneous hypoglycemia precipitated by fasting [13, 14]. One child in our study who did not have GH tested at the time of hypoglycemia was soon diagnosed with severe GH deficiency based on poor growth (height SD − 3.2) and a very low IGF-1 and IGF-binding protein 3. Another patient initially thought to have GH deficiency based on a low GH after a 12 h fast and a subnormal stimulated GH level failed to have resolution of hypoglycemia during GH therapy and was taken off treatment with normal growth. It thus is unlikely that GH levels obtained as part of the evaluation of KH are helpful in the setting of normal linear growth.

Cortisol levels are obtained for the purpose of excluding primary or secondary adrenal insufficiency (AI). However, like GH, random cortisol levels obtained during spontaneous hypoglycemia are often subnormal. One study found that 61% of 76 children who underwent a fasting study had a peak cortisol of < 18 μg/dL (497 nmol/L), which is the standard based on the threshold response to insulin-induced hypoglycemia, and subnormal responses were found in congenital hyperinsulinism, KH, and children who tolerated an age-appropriate duration of fasting normally. We considered 10 μg/dL (276 nmol/L) as a cortisol level high enough to make severe AI unlikely and found 84% had a cortisol above this level, but it was clear from our chart reviews that many of the cortisol levels were not done at the time of hypoglycemia. Primary adrenal insufficiency is rare in children except for congenital adrenal hyperplasia which presents in infancy with genital abnormalities or a salt-losing crisis [15]. Autoimmune AI generally presents in older children with long-term symptoms including weakness, weight loss, nausea, abdominal pain, and increased pigmentation, and hyponatremic dehydration at diagnosis. Secondary adrenal insufficiency most often co-exists with GH deficiency, which increases the risk of hypoglycemia, so in a normally growing child, it is a very unlikely diagnosis. Thus, the value of obtaining cortisol levels during an admission for hypoglycemia in a previously healthy child is low, though a level of > 18 μg/dL (497 nmol/L) conclusively rules out AI.

The value of ordering plasma amino acids and urine organic acids, which in our patients was done in 58%, especially when the Genetic service was consulted, is likewise low in typical cases of KH. Several organic acidemias can be diagnosed with these tests, including those involving propionic, methylmalonic, glutaric and isovaleric acids. However, these conditions usually present in very sick infants in the first weeks to months of life with metabolic acidosis and are unlikely to be found in the typical young child who is healthy until an episode of hypoglycemia following a period of poor oral intake. While abnormal urine organic acid profiles were identified in several patients, follow-up genetic consultations indicated that these were likely secondary to KH and not primary abnormalities. There was a single case in which glutaric aciduria was initially suspected but later ruled out based on repeat urine testing and genetic testing. A diagnostic fast, done in 27% of our patients, also did not appear to be helpful in that it was either normal or confirmed a diagnosis of KH if hypoglycemia with urine ketones or elevated serum β hydroxybutyrate were found.

More extensive genetic evaluation did prove to be useful in very selected cases, including the child diagnosed with GSD type 9α and the one with a very rare ketone utilization defect due to a mutation in the MCT-1 gene, but both had clinical courses atypical for idiopathic KH. The child who was ultimately diagnosed with RSS by genetic testing had severe short stature and low BMI with a prominent forehead which ultimately led to the diagnosis. A study which looked at 24 children with RSS under age 4 who had suspected or documented hypoglycemia, many of whom were described as picky eaters, found that 7 became hypoglycemic during a supervised fast of 3–18 h. This suggests that RSS children, who often have little subcutaneous fat, are prone to hypoglycemia if not fed regularly and that parents should be advised to avoid prolonged fasting [12].

While it is recognized that idiopathic KH is a diagnosis of exclusion with a fairly typical age at presentation and clinical course, it is still not known what makes children with idiopathic KH less tolerant of prolonged fasting than other children, though even normal children in the same age range may develop borderline hypoglycemia during a 24 h fast [3, 4]. It is possible based on an earlier study that they are less able to switch to gluconeogenesis from muscle protein after glycogen stores are depleted during a fast [5], but no specific defect has been identified. It is also not clear why children almost always outgrow this condition by age 6.

Strengths of our study are that it is by far the largest series of KH patients, and we were able to confirm the low frequency of other diagnoses based on long-term follow-up information in the HER plus parent interviews for about 2/3 of the patients. Limitations include the fact that not all of the laboratory results we reviewed were obtained at the time of hypoglycemia, and we may have missed some relevant laboratory testing performed outside our institution. Also, we were unable to contact some parents by mail or by phone, so important diagnoses not found in the EHR could have been missed, even though none were found in the children whose parents we did contact.

## Conclusions

1. A careful history, exam, and limited laboratory testing obtained at the time of the initial episode will identify most children with idiopathic KH. Most but not all have a history of poor intake often associated with a GI illness but about a quarter may have no such history. Documentation of either urine ketones as close as possible to the time of hypoglycemia or a serum β hydroxybutyrate as well as a serum CO_2_, which is frequently low but not < 12 mmol/L are usually sufficient.
2. Growth hormone and cortisol levels are often low following spontaneous hypoglycemia and can be omitted unless short stature or signs suggestive of cortisol deficiency are present. Insulin levels are not useful unless the hypoglycemia is non-ketotic or hypoketotic.
3. Plasma amino and urine organic acids as well as fasting studies are unlikely to aid in the diagnosis. However, referral for a genetic consultation is important if episodes of hypoglycemia are unusually frequent or severe, though up to 4–5 episodes are not uncommon. GSD is occasionally found in such patients, especially GSD 9α.
4. It is important to educate parents as to the likely etiology of their child’s hypoglycemia and counsel them on avoidance of prolonged fasting as it may both prevent further episodes and allow some of these episodes to be treated by the parents at home.

## Supplementary information

**Additional file 1.** Chart reviews including demographic, clinical, and laboratory data and follow-up of patients with ketotic hypoglycemia.

## Data Availability

The spreadsheet used for data collection and analysis has been uploaded as a supplement to the manuscript with identifying patient information removed.

## Abbreviations

AI: Adrenal insufficiency
BG: Blood glucose
ED: Emergency department
EHR: Electronic health record
GH: Growth hormone
GSD: Glycogen storage disease
IGF-1: Insulin-like growth factor 1
KH: Ketotic hypoglycemia
MCT-1: Monocarboxylate transporter 1
RSS: Russell Silver syndrome
